# Health Impacts of the Built Environment: Within-Urban Variability in Physical Inactivity, Air Pollution, and Ischemic Heart Disease Mortality

**DOI:** 10.1289/ehp.1103806

**Published:** 2011-10-17

**Authors:** Steve Hankey, Julian D. Marshall, Michael Brauer

**Affiliations:** 1 Department of Civil Engineering, University of Minnesota, Minneapolis, Minnesota, USA; 2 School of Population and Public Health, University of British Columbia, Vancouver, British Columbia, Canada

**Keywords:** active travel, air quality, environmental planning, infill, risk assessment, urban form

## Abstract

Background: Physical inactivity and exposure to air pollution are important risk factors for death and disease globally. The built environment may influence exposures to these risk factors in different ways and thus differentially affect the health of urban populations.

Objective: We investigated the built environment’s association with air pollution and physical inactivity, and estimated attributable health risks.

Methods: We used a regional travel survey to estimate within-urban variability in physical inactivity and home-based air pollution exposure [particulate matter with aerodynamic diameter ≤ 2.5 μm (PM_2.5_), nitrogen oxides (NO_x_), and ozone (O_3_)] for 30,007 individuals in southern California. We then estimated the resulting risk for ischemic heart disease (IHD) using literature-derived dose–response values. Using a cross-sectional approach, we compared estimated IHD mortality risks among neighborhoods based on “walkability” scores.

Results: The proportion of physically active individuals was higher in high- versus low-walkability neighborhoods (24.9% vs. 12.5%); however, only a small proportion of the population was physically active, and between-neighborhood variability in estimated IHD mortality attributable to physical inactivity was modest (7 fewer IHD deaths/100,000/year in high- vs. low-walkability neighborhoods). Between-neighborhood differences in estimated IHD mortality from air pollution were comparable in magnitude (9 more IHD deaths/100,000/year for PM_2.5_ and 3 fewer IHD deaths for O_3_ in high- vs. low-walkability neighborhoods), suggesting that population health benefits from increased physical activity in high-walkability neighborhoods may be offset by adverse effects of air pollution exposure.

Policy implications: Currently, planning efforts mainly focus on increasing physical activity through neighborhood design. Our results suggest that differences in population health impacts among neighborhoods are similar in magnitude for air pollution and physical activity. Thus, physical activity and exposure to air pollution are critical aspects of planning for cleaner, health-promoting cities.

Physical inactivity is associated with increased risk of several adverse health outcomes including heart disease, type 2 diabetes, colon cancer, breast cancer, and mortality (Colditz et al. 1997; Kelley and Goodpaster 2001; Kohl 2001; Verloop et al. 2000). Active commuting, such as walking or biking to work on a daily basis, has been shown to decrease risk of all-cause mortality and cardiovascular disease (Andersen et al. 2000; Hamer and Chida 2008; Zheng et al. 2009). Various attributes of the built environment (e.g., population density, street connectivity, land use mix) have been associated with rates of physical activity at the neighborhood level (Ewing et al. 2003; Frank et al. 2005; Saelens et al. 2003a; Sallis et al. 2009). Furthermore, the type of transportation mode used (public transit vs. car) affects personal energy expenditure (Morabia et al. 2010). Thus, an important research question is whether urban planning can reduce physical inactivity and improve health.

Exposure to outdoor urban air pollution is associated with various adverse health outcomes including heart disease, respiratory disease, lung cancer, asthma, and mortality (Brunekreef and Holgate 2002; Gent et al. 2003; Pope and Dockery 2006; Pope et al. 2002). Chronic exposures vary at similar magnitudes within-cities as between-cities (Jerrett et al. 2005; Miller et al. 2007), suggesting that neighborhood location, urban design, and proximity to roads can affect exposures (Health Effects Institute 2009; Marshall et al. 2005).

Recently, the World Health Organization (WHO) cited physical inactivity (4th) and exposure to outdoor urban air pollution (14th) among the top 15 risk factors for the Global Burden of Disease (WHO 2009); for high-income countries, these ranks are 4th (physical inactivity) and 8th (outdoor air pollution). Urban planning and the built environment may differentially influence exposures to those two risk factors (Marshall et al. 2009). A small number of studies have investigated the effects of exercise while controlling for air pollution exposure (de Nazelle et al. 2009; Wong et al. 2007) or explored regional- or national-scale theoretical shifts to active travel (de Hartog et al. 2010; Grabow et al. 2011); however, accounting for health outcomes from exposure to air pollution and physical inactivity among neighborhood types is a little-studied area.

We used risk assessment to explore urban-scale spatial patterns in exposures associated with the built environment. We investigated differences in urban form that have been associated with physical inactivity and air pollution [specifically, particulate matter with aerodynamic diameter ≤ 2.5 μm (PM_2.5_), nitrogen oxides (NO_x_), and ozone (O_3_)] to assess relationships between urban form and public health.

## Methods

**Figure 1:**
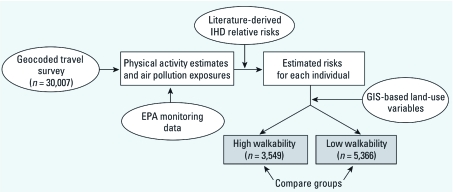
Conceptual framework for this risk assessment. Ovals are inputs, and boxes are midpoint calculations. Shaded boxes indicate estimated risk separated into two groups for comparison.

Our approach combined four primary sources of information: a geocoded, self-report travel diary to indicate home location and physical activity levels for a specific cohort (*n* = 30,007); modeled and measured estimates of outdoor air pollution concentrations and their variability in space and time; literature-derived estimates relating ischemic heart disease (IHD) rates with physical inactivity and exposure to air pollution; and geographic information system (GIS) land use variables related to walkability. Our method is descriptive (i.e., cross-sectional) and aims to explore long-term health effects of neighborhood characteristics and location. Figure 1 illustrates our risk assessment approach.

*Physical inactivity and air pollution exposures.* We used the year 2001 Post-Census Regional Travel Survey to estimate exposure to physical inactivity and home-based exposure to outdoor air pollution. This survey, which covers southern California communities such as Orange County and Los Angeles, included a geocoded time–activity diary that captured self-reported activities and travel during fall 2001 and spring 2002. The survey population consisted of a random sample of residents, recruited by telephone in six southern California counties [Imperial, Los Angeles, Orange, Riverside, San Bernardino, and Ventura; for survey details, see Southern California Association of Governments (SCAG 2003)]. To our knowledge, no other metropolitan-scale travel survey has been used to estimate physical activity and exposure to air pollution (Marshall et al. 2006); in addition, this survey represents one of the largest exposure-relevant surveys available for any urban area in the world.

Of the 40,376 survey respondents, 30,007 (74%) met our inclusion criteria: *a*) geocoded home location [2,346 respondents excluded (5.8%)], *b*) home location within the air pollution modeling domain—the South Coast Air Basin [4,491 respondents excluded (11.1%)], and *c*) complete demographic information [age, sex, and ethnicity; 3,532 respondents excluded (8.7%)]. The survey generally covered 1 weekday per participant. We multiplied each participant’s 1-day physical activity record by 7 to obtain an estimate of weekly minutes of physical activity. This approach assumed that physical activity was constant across all days of the week. Population-average levels of physical activity were similar (< 15% difference) between weekdays and weekends (11 vs. 12 min/day, respectively) based on data from a small number of respondents (13%, *n* = 5,104) who participated in an additional weekend survey supplement (see sensitivity analysis 1, below). The survey recorded total physical activity and separately disaggregated that total into active transport (e.g., walking, bicycling) versus recreational activities (e.g., sports, working out at a gym).

Our primary estimates for air pollution exposure were based on monitoring data [U.S. Environmental Protection Agency (EPA) 2010] for PM_2.5_, NO_x_, and O_3_ in 2001. We interpolated concentrations [inverse-distance weighted average of the nearest three monitors (Marshall et al. 2008)] to each survey participant’s home location. Each pollutant had several monitoring stations (PM_2.5_, 27; NO_x_, 42; O_3_, 52), providing good spatial coverage for the 36,000-km^2^ study area. We estimated the annual average of daily 1-hr maximum concentrations for O_3_ and annual-average concentrations for PM_2.5_ and NO_x_ at each survey participant’s residence to match the metrics used in the epidemiological studies that we used to estimate IHD risks. We used spatial interpolation for the base case because it can be used for all three pollutants and is easily transferable to other urban areas.

*Neighborhood walkability.* We calculated three built environment variables to represent neighborhood type: *a*) population density, *b*) intersection density, and *c*) land use mix. Neighborhoods that were in the upper (lower) tertile of all three built environment variables were defined as high- (low-) walkability neighborhoods. This approach classified 12% of the survey population as living in a high-walkability neighborhood and 18% as living in a low-walkability neighborhood. We used objective measurements of the built environment rather than geographical overlays to match methods commonly used in the urban planning literature. Although no standard measure of walkability exists, most indices include measures of density, connectivity, and land use mix (Ewing and Cervero 2001). As a sensitivity analysis, based on prior research (Marshall et al. 2009) we implemented a second definition that classified 33% of survey participants in high- and 33% in low-walkability neighborhoods [for methods, see Supplemental Material, p. 2 (http://dx.doi.org/10.1289/ehp.1103806)]. Results were similar for both definitions; therefore, we report results using the first definition only.

Population density. We used U.S. Census data from the year 2000 to calculate population density at the tract level for each household (U.S. Census Bureau 2000). Population density has been shown to be a predictor of per capita automobile travel (Holtzclaw et al. 2002; Marshall 2008) and trip length (Ewing and Cervero 2001), both of which are predictors of bicycling and walking (Handy et al. 2002).

Intersection density. Intersection density was calculated using road TIGER/Line data (U.S. Census Bureau 2000). A 1-km non-freeway network buffer was generated for each household using ArcGIS (version: 9.3.1, ESRI; Redlands, CA, USA). Intersections (more than two road segments) were summed within the buffer, yielding a measure of street connectivity. Previous studies show that street connectivity may reduce vehicle travel and increase walking (Ewing and Cervero 2001; Forsyth et al. 2008).

Land use mix. Following Frank et al. (2004), we calculated a land use mix index for each household location. Aerial land use data was obtained from SCAG for the year 2001 (SCAG 2010). The index [see Supplemental Material, pp. 2–3 (http://dx.doi.org/10.1289/ehp.1103806)] is a normalized ratio of the mix of four primary land uses (residential, commercial, retail, and institutional) to total land area within the 1-km network buffer. The index ranges from 0 to 1: A value of 1 represents an equal mixture of the four land uses; a value of 0 indicates 100% of land is a single land use. Impacts of land use mix on health include reducing obesity (Frank et al. 2005) and increasing physical activity (Saelens et al. 2003b).

*Dose–response and relative risk estimates.* For each survey participant (i.e., at the individual level), we estimated relative risks (RRs) attributable to outdoor air pollution and physical inactivity for one important health outcome: IHD. IHD is consistently associated with outdoor air pollution and physical inactivity (WHO 2009), is responsible for a large proportion of deaths in the United States (~ 18% of all deaths and 67% of heart disease deaths in 2006) [Centers for Disease Control and Prevention (CDC) 2009], and has been shown to be an important health outcome for both risk factors when considering large-scale shifts to active travel (Woodcock et al. 2009). Because our exposure estimates for air pollution are continuous, we estimated an RR for each survey participant based on a linear dose–response [see Supplemental Material, Figure S2 (http://dx.doi.org/10.1289/ehp.1103806)] for the range of observed air pollutant concentrations and the referent exposure levels described below. In contrast, WHO (2004) suggests a three-tier dose–response for physical activity: *a*) active (exercise for > 150 min/week; RR = 1), *b*) insufficiently active (exercise for 1–150 min/week; RR = 1.31), and *c*) inactive (0 min exercise per week; RR = 1.47), allowing for only three possible physical activity RRs for each survey participant. We estimated attributable fractions for outdoor air pollution and physical inactivity using the mean individual RR in high- or low-walkability neighborhoods.

Air pollution dose–response relationships were identified and selected as follows. We manually searched the tables of contents of four journals (*Journal of the American Medical Association*, *New England Journal of Medicine*, *British Medical Journal*, *Lancet*) for the years 2000–2010 for air pollution risk estimates. We also performed a search of key words in Google Scholar and ISI Knowledge, including (in various combinations) “air pollution,” “O_3_/NO_x_/PM_2.5_,” “ischemic heart disease,” “cardiovascular disease,” “cardiopulmonary disease,” “respiratory disease,” “mortality,” “health effects,” “chronic/acute,” and “dose–response.” We used the “cited by” function in Google Scholar to explore subsequent studies related to each article. Through this process, we identified 62 articles. We then selected studies that focused on within-city variation and included IHD as a health outcome (Table 1).

**Table 1:** Summary of RR estimates used for IHD.

Study	Risk factor	Study details	RR (95% CI)
Nafstad et al. 2004		NO_x_		Within-city; men 40–49 years of age in Oslo, Norway (*n* = 16,209)		1.08*a* (1.06, 1.11) per 10 μg/m^3^
Jerrett et al. 2005		PM_2.5_		Within-city; subset (Los Angeles, CA) of the ACS cohort (*n* = 22,905)		1.25*a* (0.99, 1.59) per 10 μg/m^3^
Jerrett et al. 2009		O_3_		Between-cities; ACS cohort (*n* = 448,850)		1.008*a* (1.002, 1.013) per 10 μg/m^3^
WHO 2004		Physical inactivity		Meta-analysis of 20 studies from two continents (Western Europe, 8; North America, 12; total *n* = 327,004 )		Insufficiently active:*b* 1.31 (1.21, 1.41) Inactive:*b* 1.47 (1.39, 1.56)
**a**Air pollution risk estimates used here were based on long-term cohort studies and chronic health effects. **b**Referent, > 150 min/week; insufficiently active, 1–150 min/week; inactive, 0 min/week.						

Each RR for air pollution was estimated from cohort studies of long-term exposures; however, these estimates differed in important ways. For example, Nafstad et al. (2004) studied men 40–49 years of age, meaning our NO_x_ results cannot be generalized to other populations [RR = 1.08; 95% confidence interval (CI): 1.06, 1.11]. Jerrett et al. (2005) used a subset of the American Cancer Society (ACS) cohort (Los Angeles, CA, USA) to estimate a within-city RR of 1.25 per 10 μg/m^3^ increase in PM_2.5_ (95% CI: 0.99, 1.59). Jerrett et al. (2005) did not report a significant RR for PM_2.5_ in Los Angeles, but the RR estimate is roughly consistent with two between-city studies that did report statistically significant RRs: Pope et al. (2004; RR = 1.18 per 10 μg/m^3^ increase in PM_2.5_; 95% CI: 1.14, 1.23) and Jerrett et al. (2009; RR = 1.21; 95% CI: 1.16, 1.27). The Jerrett et al. (2009) RR for a 10 μg/m^3^ increase in O_3_ (1.008; 95% CI: 1.002, 1.013) was based on between-city variation (ACS cohort) in 96 U.S. metropolitan statistical areas generated from a one-pollutant model. However, it is important to note that Jerrett et al. (2009) reported a protective effect for O_3_ based on a two-pollutant model adjusted for PM_2.5_ (RR = 0.97; 95% CI: 0.96, 0.99), and overall there is less evidence in the literature for O_3_ associations with IHD compared with those for PM_2.5_. A within-city study of O_3_ and IHD was not available.

The referent exposure levels used to estimate individuals’ RRs were “active” for physical inactivity (> 150 min of moderate-vigorous activity per week), and the 10th percentile of exposure (survey population based; values: 13.6 μg/m^3^ for PM_2.5_, 39.8 μg/m^3^ for NO_x_, 80.3 μg/80.3 μg/m^3^ for O_3_) for air pollution, consistent with exposures in a relatively clean neighborhood in the study area. Each survey participant’s air pollution RR was estimated based on the difference between their home-location air pollution exposure and the referent exposure level. For example, for PM_2.5_, an individual whose home-location exposure estimate was 23.6 μg/m^3^ (10 μg/m^3^ above the referent level) would be assigned an RR of 1.25.

*Population-attributable fraction.* We calculated population-attributable fraction (PAF) and estimated attributable IHD mortality rates for each risk factor in high- and low-walkability neighborhoods. PAF for a neighborhood was calculated based on the proportion of individuals exposed to each risk factor and average RR among all individuals in a neighborhood (Baker and Nieuwenhuijsen 2008):


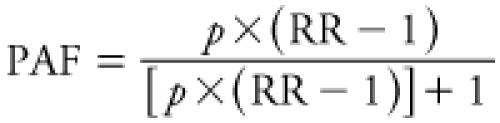
[1]

Here, RR is the mean individual RR in each group (high- and low-walkability neighborhoods) and risk factor, and *p* is the proportion of individuals exposed in each group (defined by our referent exposure levels). We used the 2000–2001 age-adjusted IHD mortality rate in California (191.2 IHD deaths/100,000/year; CDC 2011) to estimate deaths within each group and subsequent attributable IHD mortality rates (except for NO_x_ where we used the IHD mortality rate for men in California 45–54 years of age: 81.9 IHD deaths/100,000/year). Attributable mortality due to physical inactivity, PM_2.5_, NO_x_, and O_3_ cannot be summed because of confounding among the risk factors and overlap of at-risk populations. Therefore, we report attributable mortality due to the different factors separately.

We separately calculated PAF using a method with multiple exposure levels instead of the dichotomous exposure levels implicit in Equation 1, as described in the Supplemental Material [pp. 5–6 (http://dx.doi.org/10.1289/ehp.1103806)] Results based on this alternative method were similar to those reported below.

*Sensitivity analyses.* To explore the robustness of our estimates, we used three sensitivity analyses to assess *a*) different methods of scaling minutes of physical activity, *b*) alternate modeling approaches for air pollution, and *c*) stepwise versus linear dose–response for physical activity.

Sensitivity analysis 1: scaling method for minutes of physical activity. Our approach requires extrapolating weekly exercise rates based on the 1-day travel diary because most physical activity epidemiological literature employs the metric “minutes of physical activity per week.” To test the limitations of this extrapolation for our analysis, we developed a Monte Carlo simulation that relaxes our base-case assumption (i.e., that individuals’ physical activity rates are constant by day), by employing two alternative assumptions: that people who are nonsedentary are physically active *a*) every other day or *b*) every third day. The Monte Carlo simulation distributes total minutes of physical activity accordingly, stratifying by age, sex, and ethnicity. The resulting distributions of physical activity better approximate national estimates on the prevalence of physical inactivity (WHO 2004).

Sensitivity analysis 2: air pollution model. Our base-case analysis used spatial interpolation of U.S. EPA monitoring data, which are readily available for all three pollutants for many urban areas. We compared results using a Eulerian dispersion model [Comprehensive Air Quality Model with Extensions (CAMx); http://www.camx.com; nitrous oxide (NO), nitrogen dioxide (NO_2_), O_3_] and land-use regression (LUR; NO_2_; Novotny et al. 2011). CAMx and LUR provide greater spatial precision than inverse-distance weighting but may or may not be available in other urban areas.

Sensitivity analysis 3: physical activity dose–response. We tested the sensitivity of our results to the dose–response curve for physical inactivity. Our base case used the stepwise dose–response from WHO (2004) (Table 1). For this sensitivity analysis, we generated three linear dose–response curves (low, medium, and high slopes) based on the same WHO values.

## Results

Annual-average air pollution exposure for the survey population averaged 49 μg/m^3^ for NO_2_ [interquartile range (IQR), 41–60 μg/m^3^), 99 μg/m^3^ for O_3_ (86–112 μg/m^3^; annual average of 1-hr daily maximums), and 22 μg/m^3^ for PM_2.5_ (20–24 μg/m^3^; Table 2). Mean NO_2_ exposures were below current ambient-air standards [U.S. EPA and California Environmental Protection Agency (CalEPA) standards, respectively: 100 and 57 μg/m^3^]. PM_2.5_ exposures were approximately 1.5 and 2 times higher than U.S. EPA (15 μg/m^3^) and CalEPA (12 μg/m^3^) long-term standards (annual arithmetic mean), respectively (California Air Resources Board 2010).

**Table 2:** Descriptive statistics by neighborhood type [mean (IQR)].

Variable	All (*n* = 30,007)	Low walkability (*n* = 5,366)	High walkability (*n* = 3,549)
Age (years)		38 (21–54)		41 (23–58)		34 (20–47)
Nonwhite (%)		40		23		65
Male (%)		50		49		50
Income > $50,000 per year (%)		48		57		31
College or more (%)		46		52		40
NO_x_ (μg/m^3^)*a*		85 (68–103)		67 (50–88)		106 (89–130)
O_3_ (μg/m^3^)*b*		99 (86–112)		111 (97–124)		86 (82–92)
PM_2.5_ (μg/m^3^)*a*		22 (20–24)		20 (14–25)		23 (22–24)
Physical activity (min/week)		77 (0–0)		68 (0–0)		102 (0–0)
Population density in Census tract (people/km^2^)*c*		22,400 (7,800–28,400)		3,100 (600–5,200)		53,500 (31,900–61,600)
Intersection density (1-km network buffer)*c*		57 (27–82)		11 (2–20)		109 (86–114)
Land use mix (1-km network buffer)*c*		0.37 (0.25–0.49)		0.13 (0–0.23)		0.59 (0.50–0.66)
All continuous variables in high-walkability neighborhoods have statistically significant differences (for all variables *p *< 0.001) compared with low-walkability neighborhoods (two-tailed *t*-test). **a**Home-location annual-average concentrations. **b**Home-location annual average of daily 1-hr maximum concentrations. **c**This land use variable was used to define walkability.						

Self-reported physical activity levels averaged 77 min/week (IQR, 0–0 min/week; i.e., the 25th and 75th values are 0 min/week; Table 2). Most (83.5%) of the survey participants reported being inactive (0 min/week), 5.6% reported being insufficiently active (1–150 min/week), and 10.9% reported being active (> 150 min/week; physical activity recommendations; U.S. Department of Health and Human Services 1996). Activity levels were notably lower than national averages (U.S. averages: inactive, 29%; insufficiently active, 45%; active, 26%; WHO 2004). Sensitivity analysis 1 addresses this difference in activity levels.

**Figure 2:**
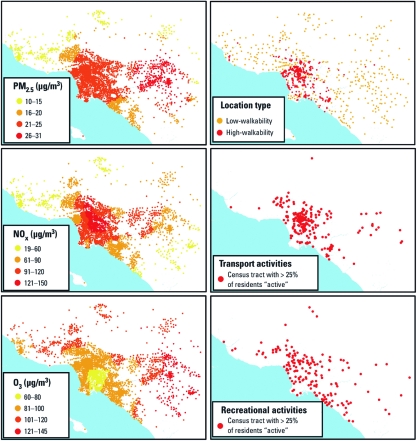
Spatial variation of air pollution exposure and physical inactivity. Physical activity estimates were derived from time–activity diaries, air pollution exposures were calculated from U.S. EPA monitoring data, and walkability was defined using publicly available land use variables. Icons for transport and recreational activities represent census tracts where > 25% of the survey respondents reported > 150 min/week of that activity type.

NO_x_ and PM_2.5_ concentrations were highest near the city center and major roadways, whereas O_3_ concentrations were higher in the outer-lying areas (Figure 2). Because of this spatial pattern, few locations experienced low exposure to all three pollutants. Spatial patterns for physical activity were dependent on the purpose of the activity; there was no discernable spatial pattern for recreational activities, but active transport was clustered near high-walkability neighborhoods (Figure 2).

**Figure 3:**
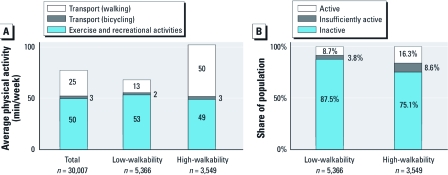
Differences among neighborhoods. (*A*) Average active transport (minutes walking and bicycling per person) and recreational activities. (*B*) Physical activity levels. The between-neighborhood difference in total physical activity is statistically significant (*p* < 0.001, two-tailed *t*-test).

Average per capita physical activity was 50% higher in high- than in low-walkability neighborhoods (102 vs. 68 min/week; Figure 3). The number of nonsedentary individuals (people with > 0 min/week physical activity) was two times higher in high- versus low-walkability neighborhoods (24.9% and 12.5%, respectively; *p* < 0.001). However, considering nonsedentary individuals only, average physical activity was 24% lower in high- than in low-walkability neighborhoods (410 vs. 543 min/week). This finding suggests that neighborhood type may have differing impacts on the number of people participating in physical activities, average physical activity among all individuals, and average physical activity among nonsedentary individuals.

The self-reported purpose of physical activity differs by neighborhood (Figure 3). For example, active transport accounts for about half of physical activity in the high-walkability neighborhoods but only 20% in low-walkability neighborhoods. Active transport is 3.6 times higher in high- versus low-walkability neighborhoods (a finding that partially corroborates our GIS estimates of walkability), whereas nontravel activity is similar (< 10% difference) in low- versus high-walkability neighborhoods. Activity level and purpose exhibited greater weekend/weekday differences in low-walkability areas than in high-walkability areas [see Supplemental Material, Table S2, Figure S4 (http://dx.doi.org/10.1289/ehp.1103806)].

**Figure 4:**
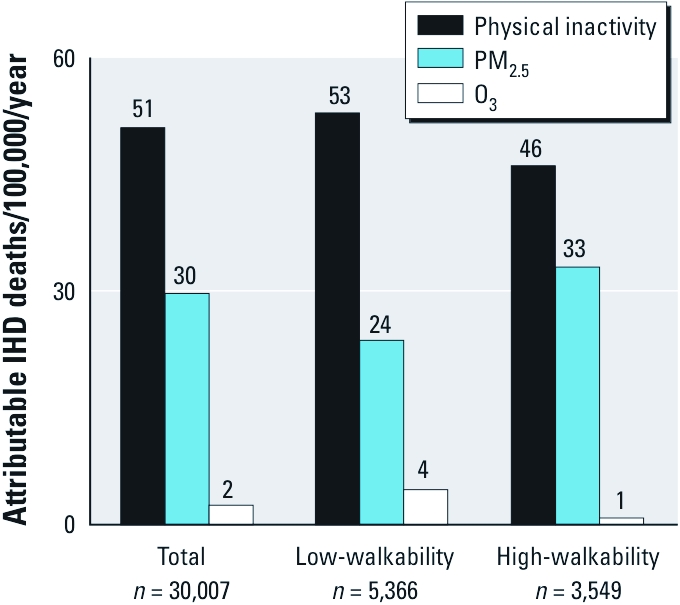
Estimated attributable IHD mortality rates for each risk factor and neighborhood type. Rates were calculated using means of individual RRs and prevalence of exposure within neighborhood type [referent, > 150 min/week of physical activity; 10th percentile of air pollution exposure (13.6 μg/m^3^ for PM_2.5_, 39.8 μg/m^3^ for NO_x_, and 80.3 μg/m^3^ for O_3_)]. The overall incidence of IHD mortality in California is 191 deaths/100,000/year (CDC 2011).

Figure 4 shows estimated attributable IHD mortality rates for each neighborhood type and risk factor. Physical inactivity was more strongly associated with IHD mortality (51 additional deaths/100,000/year overall) than were the other exposures, but IHD mortality attributable to physical inactivity was only slightly different between high- and low-walkability neighborhoods (7 fewer IHD deaths/100,000/year in high- vs. low-walkability). Conversely, overall estimated attributable IHD mortality due to exposure to PM_2.5_ was smaller (30 deaths/100,000/year), but the difference between neighborhoods was slightly larger than for physical inactivity (9 more IHD deaths/100,000/year in high- vs. low-walkability). O_3_ shows the reverse spatial pattern as PM_2.5_ (i.e., O_3_ exposure is higher in low-walkability neighborhoods, whereas PM_2.5_ is lower) but a smaller difference in mortality between neighborhoods (3 fewer IHD deaths/100,000/year in high- vs. low-walkability). Attributable IHD mortality rates for NO_x_ (represented by risk estimates for men 40–49 years of age; not shown in Figure 4) were 13 (28) IHD deaths/100,000/year for low- (high-) walkability neighborhoods. Attributable risk estimates for physical inactivity, PM_2.5_, and O_3_ showed similar patterns when neighborhoods were classified according to deciles of walkability scores [Supplemental Material, Figure S5 (http://dx.doi.org/10.1289/ehp.1103806)].

*Sensitivity analysis 1: scaling method for minutes of physical activity.* Results [see Supplemental Material, pp. 8–9 (http://dx.doi.org/10.1289/ehp.1103806)] indicate that our alternative assumptions reduce the variability in physical activity among neighborhoods. Specifically, the Monte Carlo simulation increases the share of nonsedentary individuals (subsequently reducing average risks from physical inactivity) but also yields reductions in estimated IHD mortality differences among neighborhoods. Our core conclusions are similar among the Monte Carlo simulations.

*Sensitivity analysis 2: air pollution model.* Central tendencies varied by pollutant and model; however, trends in the core conclusions (i.e., shifts in exposure and risk by neighborhood type) were similar where it was possible to compare [see Supplemental Material, pp. 9–10 (http://dx.doi.org/10.1289/ehp.1103806)]. In general, differences in estimated IHD mortality rates between high- and low-walkability neighborhoods were larger when using the alternate models; therefore, base-case results reported above may be conservative estimates (i.e., underestimates) of air pollution spatial variability.

*Sensitivity analysis 3: physical activity dose–response.* Our results did not change appreciably when using the linear dose–response curves [see Supplemental Material, pp. 10–11 (http://dx.doi.org/10.1289/ehp.1103806)].

We also estimated RRs according to neighborhood type (high- or low-walkability) within strata of age (0–25 years, 26–50 years, > 50 years) and according to income and ethnicity [high income (> $75,000) and white vs. low income (< $35,000) and nonwhite]. The results reveal similar trends in risk differences between neighborhoods for each strata, suggesting that our results are robust to accounting for differences in income, ethnicity, and age. Details are in the Supplemental Material [pp. 11–14, Table S6 (http://dx.doi.org/10.1289/ehp.1103806)]. Prior literature further explores socioeconomic aspects of this topic (e.g., Ewing 2005; Frank et al. 2007; Sallis et al. 2009).

## Discussion

Our analysis summarizes between-neighborhood variations in two risk factors (exposure to air pollution, physical inactivity) using a time–activity travel diary for one region. We found risks were differential when stratified by neighborhood walkability. Specifically, when comparing estimated IHD mortality rates among neighborhoods, differences attributable to physical inactivity were modest and comparable to differences attributable to individual air pollutants. Because of spatial patterns associated with each pollutant, urban residents were often highly exposed to at least one but not all pollutants (e.g., high exposure to O_3_ in low-walkability neighborhoods or high exposure to PM_2.5_ in high-walkability neighborhoods). This trade-off suggests that the net health impact of neighborhoods may depend in part on spatial patterns of air pollution.

Recent health comparisons between air pollution and exercise (Carlisle and Sharp 2001; de Hartog et al. 2010) emphasize the greater health importance of exercise relative to air pollution. This prior research considered only people who exercise (Carlisle and Sharp 2001; de Hartog et al. 2010); here, we consider the entire population—nonsedentary plus sedentary individuals. Only a subset of a given population is physically active, and only a subset of that physical activity is influenced by neighborhood design; here, the net result is that spatial differences in attributable IHD mortality risks are of similar magnitude for physical inactivity as for air pollution. Our results indicate a doubling in the share of nonsedentary people in high- versus low-walkability neighborhoods (24.9% vs. 12.5%); however, all individuals—inactive and active—experience changes in air pollution exposures. For this study population, physical activity rates were higher (and exercise-attributable IHD mortality rates lower) in high- than in low-walkability neighborhoods. However, because variations in air pollution risk are similar to variations in physical inactivity risks, when comparing high- versus low-walkability neighborhoods, health benefits from increased physical activity may be offset by health risks from air pollution exposure.

Our study uses self-reported rather than objectively measured physical activity. Previous studies that have used objectively-measured physical activity to investigate effects of urban form on physical activity (Table 3) have reported mixed results: two studies reported differences in physical activity by neighborhood type (Frank et al. 2005; Sallis et al. 2009), and one indicated shifts in the purpose (transport vs. fitness) but not the amount of physical activity (Forsyth et al. 2008). These findings suggest that urban-scale differences in physical activity rates are similar between objectively measured physical activity and our self-reported measures of activity. For example, differences in per capita physical activity between high- and low-walkability neighborhoods in Seattle, Washington, and Baltimore, Maryland, were similar to differences in our southern California population [41 min/week (Seattle, Baltimore) versus 34 min/week (southern California) (Sallis et al. 2009)].

**Table 3:** Comparison of results from studies using objective measures of physical activity with results from the present study.

Study	Location	Measure of physical activity	Measure of urban form	Core result
Sallis et al. 2009		Seattle, WA, and Baltimore, MD		Objective: 7-day accelerometer		Walkability (net residential density, intersection density, land use mix, retail floor area ratio)		41 min/week increase in physical activity between high- vs. low-walkability neighborhoods
Frank et al. 2005		Atlanta, GA		Objective: 2-day accelerometer		Walkability (net residential density, intersection density, land use mix)		Two-fold increase in meeting physical activity recommendations in high- vs. low‑walkability neighborhoods
Forsyth et al. 2008		St. Paul, MN		Objective: 7-day accelerometer		Population density, block size (street pattern)		Significant increase in transport-related physical activity (high- vs. low-walkability neighborhoods) but no difference in total physical activity
Present study		South Coast Air Basin, CA		Self-report: one-day time activity diary		Walkability (population density, intersection density, land use mix)		34 min/week increase in physical activity between high- vs. low-walkability neighborhoods (2-fold increase in meeting physical activity recommendations)


Our study limitations include those associated with travel surveys and self-reported information in general. For example, travel surveys typically undercount trips by all modes (Bricka and Bhat 2006), affecting estimates of travel time (Wolf et al. 2003). The SCAG survey suggests that vehicle undercount rates may approach 20–25% but gives little information regarding nonmotorized trips (SCAG 2004). Undercount rates may be differential by trip length (SCAG 2004), mode, or neighborhood. Comparisons with studies using objectively measured physical activity (see preceding paragraph) suggest that our core findings are robust to trip undercounting and other problems with self-reported travel data.

Our work is motivated by the goal of understanding and designing clean, healthy, sustainable cities (Giles et al. 2011). Our investigation explores only one location (Los Angeles), one health outcome (IHD), one cohort, a small number of pollutants (NO_x_, PM_2.5_, O_3_), and physical inactivity. Clearly, further analyses incorporating other risk factors (e.g., noise, transport injury) linked to the built environment are warranted. Interaction between physical activity and air pollution may vary on an even smaller scale than we have investigated in the present study (i.e., within neighborhoods). Future analyses could use age-specific risks of IHD mortality for air pollution and physical inactivity. Our analysis is descriptive (i.e., cross-sectional) in nature; more research is needed to explore causality between urban form and health risks (especially for physical activity, because ambient air pollution exposure is largely determined by geographical location).

Despite these limitations, our results are relevant to health officials, sustainability scientists, and urban planners. To our knowledge, ours is the first analysis that directly compares health risks for both air pollution and physical inactivity among neighborhoods based on activity patterns for a random sample of residents in an urban area, and thus is the first to quantify relationships between urban form and the health impacts of physical activity and air pollution. We found that attributes of the built environment were associated with both air pollution exposure and physical inactivity. These results emphasize that to be health protective, neighborhoods designed to decrease risks from one factor must avoid unintentionally increasing risks from other factors.

## Conclusion

We compared the health impacts attributable to air pollution and physical inactivity among neighborhoods for one cohort (~ 30,000 individuals in Southern California). A larger proportion of our Southern California study population was classified as nonsedentary in high- versus low-walkability neighborhoods (25% vs. 13%). However, because only a small share of the total population was classified as physically active, we estimated only moderate differences in IHD mortality rates attributable to physical inactivity between neighborhood types. Spatial patterns of estimated attributable IHD mortality rates varied by pollutant: estimated mortality due to increased PM_2.5_ and NO_x_ were greater in high- than in low-walkability neighborhoods, whereas estimated IHD mortality due to increased O_3_ was greater in low- than in high-walkability neighborhoods. In general, differences in estimated IHD mortality between neighborhoods were comparable for exposure to air pollutants and physical inactivity. Our results suggest complex within-urban spatial trade-offs in health risks associated with air pollution and physical inactivity. Efforts to design healthy neighborhoods should account for many factors, including air pollution and physical inactivity, and not address one concern at the expense of others.
